# Cancer cachexia in a mouse model of oxidative stress

**DOI:** 10.1002/jcsm.12615

**Published:** 2020-09-12

**Authors:** Jacob L. Brown, Marcus M. Lawrence, Bumsoo Ahn, Parker Kneis, Katarzyna M. Piekarz, Rizwan Qaisar, Rojina Ranjit, Jan Bian, Gavin Pharaoh, Chase Brown, Fredrick F. Peelor, Michael T. Kinter, Benjamin F. Miller, Arlan Richardson, Holly Van Remmen

**Affiliations:** ^1^ Aging and Metabolism Research Program Oklahoma Medical Research Foundation Oklahoma City OK USA; ^2^ Oklahoma City VA Medical Center Oklahoma City OK USA; ^3^ Oklahoma Center for Neuroscience University of Oklahoma Health Sciences Center Oklahoma City OK USA; ^4^ Reynolds Center for Aging Research University of Oklahoma Health Sciences Center Oklahoma City OK USA

**Keywords:** Reactive oxygen species (ROS), CuZn superoxide dismutase knockout mice (Sod1KO), Lewis lung carcinoma cells (LLC), Lung cancer, Oxidative stress

## Abstract

**Background:**

Cancer is associated with muscle atrophy (cancer cachexia) that is linked to up to 40% of cancer‐related deaths. Oxidative stress is a critical player in the induction and progression of age‐related loss of muscle mass and weakness (sarcopenia); however, the role of oxidative stress in cancer cachexia has not been defined. The purpose of this study was to examine if elevated oxidative stress exacerbates cancer cachexia.

**Methods:**

Cu/Zn superoxide dismutase knockout (Sod1KO) mice were used as an established mouse model of elevated oxidative stress. Cancer cachexia was induced by injection of one million Lewis lung carcinoma (LLC) cells or phosphate‐buffered saline (saline) into the hind flank of female wild‐type mice or Sod1KO mice at approximately 4 months of age. The tumour developed for 3 weeks. Muscle mass, contractile function, neuromuscular junction (NMJ) fragmentation, metabolic proteins, mitochondrial function, and motor neuron function were measured in wild‐type and Sod1KO saline and tumour‐bearing mice. Data were analysed by two‐way ANOVA with Tukey–Kramer post hoc test when significant *F* ratios were determined and *α* was set at 0.05. Unless otherwise noted, results in abstract are mean ±SEM.

**Results:**

Muscle mass and cross‐sectional area were significantly reduced, in tumour‐bearing mice. Metabolic enzymes were dysregulated in Sod1KO mice and cancer exacerbated this phenotype. NMJ fragmentation was exacerbated in tumour‐bearing Sod1KO mice. Myofibrillar protein degradation increased in tumour‐bearing wild‐type mice (wild‐type saline, 0.00847 ± 0.00205; wildtype LLC, 0.0211 ± 0.00184) and tumour‐bearing Sod1KO mice (Sod1KO saline, 0.0180 ± 0.00118; Sod1KO LLC, 0.0490 ± 0.00132). Muscle mitochondrial oxygen consumption was reduced in tumour‐bearing mice compared with saline‐injected wild‐type mice. Mitochondrial protein degradation increased in tumour‐bearing wild‐type mice (wild‐type saline, 0.0204 ± 0.00159; wild‐type LLC, 0.167 ± 0.00157) and tumour‐bearing Sod1KO mice (Sod1KO saline, 0.0231 ± 0.00108; Sod1 KO LLC, 0.0645 ± 0.000631). Sciatic nerve conduction velocity was decreased in tumour‐bearing wild‐type mice (wild‐type saline, 38.2 ± 0.861; wild‐type LLC, 28.8 ± 0.772). Three out of eleven of the tumour‐bearing Sod1KO mice did not survive the 3‐week period following tumour implantation.

**Conclusions:**

Oxidative stress does not exacerbate cancer‐induced muscle loss; however, cancer cachexia may accelerate NMJ disruption.

## Introduction

Cancer cachexia is a muscle wasting syndrome defined by a loss of skeletal muscle mass that cannot be treated by nutritional therapies.^1^, ^2^ Cachexia occurs in up to 80% of cancer cases and is directly attributable for up to 40% of cancer‐related deaths.^1^, ^2^, ^3^ Current therapies to treat cancer cachexia are lacking; therefore, a critical need remains to understand the underlying mechanisms of cancer‐induced muscle wasting in order to develop an effective therapeutic strategy. In many cases, cancer cachexia develops in association with pre‐existing age‐related loss of muscle mass and function (i.e. sarcopenia).^4^, ^5^, ^6^ Oxidative stress is a key driver of sarcopenia and muscle dysfunction^7^, ^8^, ^9^; however, the role oxidative stress plays in cancer‐induced muscle loss is understudied.

Oxidative stress is an imbalance between the production of free radicals and the ability to neutralize these oxidizing molecules. Skeletal muscle reactive oxygen species (ROS) such as superoxide and its derivatives accumulate and damage macromolecules when they chronically exceed the reducing capacity of the cell. Damaged proteins induce an increase in activation of protein breakdown signalling, promoting muscle loss.^10^, ^11^ Tissues possess scavenging antioxidant enzymes that act to neutralize radicals and prevent oxidative stress‐mediated tissue dysfunction.^12^ However, scavenging enzymes are dysregulated in many disease conditions,^13^, ^14^ leading to oxidative stress. Both the content and the activity of many of these scavenger enzymes (including CuZnSOD) are disrupted in muscle from mice with cancer^15^; however, it is not known if loss of free radical scavenging potential contributes to muscle loss. CuZnSOD is a primarily cytosolic enzyme that acts to detoxify superoxide anion, a damaging reactive molecule formed from the reaction of electrons with oxygen. The Cu/Zn superoxide dismutase knockout (Sod1KO) mouse is a model that exhibits high levels of oxidative stress and damage due to loss of superoxide scavenging capacity and recapitulates the pathologies that occur in sarcopenia in an accelerated time frame. Specifically, Sod1KO mice show a significant loss of muscle innervation, muscle mass, and function.^9^, ^16^ The role of oxidative stress in promoting cancer cachexia is understudied; therefore, it is not known if oxidative stress exacerbates cancer cachexia or cancer mortality.

Mitochondria are a primary site for ROS production in skeletal muscle.^17^ Both cancer and oxidative stress impair mitochondrial quality and function, which leads to excess production of ROS.^18^, ^19^, ^20^ The accumulation of ROS contributes to skeletal muscle atrophy in many disease phenotypes^11^, ^21^, ^22^, ^23^, ^24^, ^25^, ^26^ via the induction of atrophy programming.^11^, ^21^, ^22^, ^23^, ^24^, ^25^, ^26^ Considering the role ROS has in promoting muscle wasting, and the noted loss of antioxidant activity and content in cachectic muscle and aged muscle,^15^, ^27^ there is a critical need to explore cancer‐induced muscle wasting in sarcopenic mice with high levels of oxidative stress.

To our knowledge, the role of cancer‐induced motor neuron dysfunction has not been explored and could be a key contributor to loss of muscle mass. Oxidative stress contributes to neuronal dysfunction via demyelination, which may promote muscle wasting.^28^, ^29^, ^30^ Oxidative stress also leads to neuromuscular junction (NMJ) impairments, which is important for the maintenance of skeletal muscle mass and function.^28^, ^29^ Sarcopenia is associated with NMJ disruption^31^; however, it is not known if cancer leads to NMJ pathology or exacerbates pre‐existing NMJ dysfunction.

To determine if oxidative stress exacerbates cancer cachexia, we implanted tumours in an established experimental model of oxidative stress, the Sod1KO mice. We hypothesized that deletion of *Sod1* would exacerbate cancer cachexia when compared with wild‐type (WT) counterparts. To test this hypothesis, we measured survivability, skeletal muscle size, skeletal muscle contractile function, skeletal muscle myofibrillar, cytosolic and mitochondrial protein turnover, mitochondrial function, metabolic proteomics, motor neuron function, and loss of innervation in tumour‐bearing WT and Sod1KO mice.

## Methods

### Animals and interventions

Animal experiments were approved by the Institutional Animal Care and Use Committees and performed at the Oklahoma Medical Research Foundation. In the current study, we have utilized the Lewis lung carcinoma (LLC) pre‐clinical model to study cancer cachexia. Experimental mice were group housed, kept on a 12:12 h light–dark cycle, and had access to standard rodent chow and water *ad libitum*. The breeding and characterization of the Sod1KO mice is described in detail elsewhere.^16^, ^32^ Blood was drawn through the aorta, and tissues were collected and snap frozen in liquid nitrogen for subsequent analyses.

### Lewis lung carcinoma growth and tumour implantation

Lewis lung carcinoma cells were grown and implanted as previously described.^19^ Briefly, LLC cells (ATCC CRL‐1642) were plated in 250 mL culture flasks in Dulbecco's modified Eagle medium supplemented with 10% foetal bovine serum plus 1% penicillin and streptomycin. Once confluent, cells were trypsinized, counted, and diluted in phosphate‐buffered saline for implantation. LLC cells were implanted into the right hind flank of the mouse. WT and Sod1KO mice were implanted with LLC cells at ~4–4.5 months of age. LLC cells were plated at passages 2–5. LLC cells were suspended in 100 μL of phosphate‐buffered saline for the injection, so control mice were injected with phosphate‐buffered saline into the hind flank of the mouse.

### *Ex vivo* extensor digitorum longus contractility

Extensor digitorum longus (EDL) contractile properties were measured as previously described.^8^, ^33^ Briefly, EDL muscle was suspended on a dual‐mode muscle lever system (300C‐LR, Aurora Scientific Inc, Aurora, Canada) and a hook in Krebs buffer. Muscles were placed at optimal length and allowed 20 min of thermoequilibration at 32°C. A supramaximal current (600–800 mA) of 0.25 ms pulse duration was delivered through a stimulator (701 C, Aurora Scientific Inc.), while train duration for isometric contractions was 300 ms. Data were recorded and analysed using commercial software (DMC and DMA, Aurora Scientific). Specific force (N/cm^2^) of EDL muscles were multiplied to the ratio of fibre length to muscle length published previously.^34^

### Histology

Quadricep muscle was immediately imbedded in optimal cutting temperature compound and snap frozen. Sections were cut at 10 μm with a Leica 3050 cryotome. Staining for haematoxylin and eosin and succinate dehydrogenase was performed as previously described.^19^, ^26^ Zeiss inverted microscope was used to image slides. Muscle fibres were circled using Image J imaging software.

### Determination of protein turnover

Protein synthesis was determined according to methods described in other studies.^35^, ^36^, ^37^ Mice received a bolus i.p. injection (~20 μL/g body weight) of 99% deuterium oxide (D_2_O) 5 days before tissue collection. Drinking water was thereafter supplemented with 8% D_2_O in drinking water until euthanasia.^35^, ^36^, ^37^ We chose to start labelling 5 days prior to euthanasia because this period corresponds to the period of time that the LLC model begins to lose muscle mass.^19^, ^26^ Approximately 50 mg of tibialis anterior (TA) muscles were powdered and fractionated according to our previously published protocols.^35^, ^36^, ^37^ TA muscle was used because gastrocnemius was processed for other experiments (respirometer, proteomics, RNA analysis, and immunoblot analysis). Briefly, skeletal muscle tissue was homogenized 1:20 in isolation buffer (100 mM KCl, 40 mM Tris HCl, 10 mM Tris base, 5 mM MgCl2, 1 mM EDTA, 1 mM Adenosine triphosphate, pH = 7.5) with phosphatase and protease inhibitors (HALT, Thermo Fisher Scientific) using a bead homogenizer (Next Advance Inc., Averill Park, NY, USA). After homogenization, subcellular fractions were isolated via differential centrifugation as previously described.^35^, ^36^, ^37^ The pentafluorobenzyl‐*N*,*N*‐di (pentafluorobenzyl) derivative of alanine was analysed on an Agilent 7890A GC coupled to an Agilent 5975C MS as previously described.^35^, ^36^, ^37^ Distilled plasma was analysed on a Liquid Water Isotope Analyser (LWIA‐45‐EP, Los Gatos Research, Inc., San Jose, CA, USA). The newly synthesized fraction (*f*) of proteins was calculated from the enrichment of alanine bound in muscle proteins over the entire labelling period, divided by the true precursor enrichment (*p*), using plasma D_2_O enrichment with mass isotopomer distribution analysis adjustment.^38^

### Modelling calculations to account for non‐steady state conditions

The period of D_2_O measurement was a period of muscle loss, which violates the steady state assumptions of isotopic labelling. To account for this non‐steady state condition, calculations were made based in our previously published work.^39^, ^40^, ^41^ CoxIV is commonly used as a surrogate marker for mitochondrial content; therefore, it was used for assessing turnover calculations.^42^, ^43^, ^44^ In brief, the mass of protein at time *t*, *P(t)*, obeys the differential equation:

(1) $$\frac{dP}{dt}=k_{\mathrm{syn}}-k_{\deg}P\left(t\right),$$

where *k*_*syn*_ is the synthesis rate, with dimensions of mass over time, and *k*_*deg*_ is the degradation constant, with dimensions of inverse time.

From the equations derived in Miller *et al*.,^39^, ^40^

(2) $$k_{\mathrm{syn}}=k_{\deg}P_{\mathrm{eq}}.$$

### Targeted quantitative mass spectrometry

We used targeted quantitative mass spectrometry to measure protein abundance as previously described.^45^ Briefly, gastrocnemius samples were homogenized in a radioimmunoprecipitation assay buffer containing 10 mM Tris‐Cl (pH 8.0), 1 mM EDTA, 1% Triton X‐100 (*v*/*v*), 0.1% sodium deoxycholate (*w*/*v*), 0.1% sodium dodecyl sulfate (*w*/*v*), 140 mM NaCl, and 1 mM phenylmethylsulfonyl fluoride, with protease inhibitor cocktail (Calbiochem Set III, EDTA‐free; EMD Millipore; Billerica, MA, USA), and Bradford assay was used to determine protein concentration. For targeted proteomic analysis, 150 μG protein was used, as previously described.^33^, ^46^

Each of the four independent variables was analysed using scikit‐learn's implementation of linear discriminant analysis (LDA), with the eigen decomposition performed via singular value decomposition. Additional information regarding the univariate statistics comparing genotypes are given in the Supporting Information, *Table* S2. The clear separation between the genotypes in LDA component 1 is associated with the knockout of SOD1, as expected; however, the knockout model induces several other alterations to protein levels, which are shown with the supplementary table. LDA component 2 is associated with a linear combination of variables for which is less amenable to separation or decomposition into univariate statistics.

### Respiration and hydroperoxide production in permeabilized fibre bundles

Skeletal muscle fibre permeabilization was performed as previously described.^11^, ^19^, ^33^ Briefly, small strips of red gastrocnemius muscle were teased to near‐single fibres in ice cold buffer X (7.23 mM K2EGTA, 2.77 mM CaK2EGTA, 20 mM imidazole, 0.5 mM DTT, 20 mM taurine, 5.7 mM ATP, 14.3 mM PCr, 6.56 mM MgCl2–^6^H2O, and 50 mM K‐MES with a pH of 7.1). These fibre bundles were then permeabilized with saponin for 30 min. Mitochondrial oxygen consumption rate (OCR) and hydroperoxide production rate were simultaneously measured using the Oxygraph‐2k (O2k, OROBOROS Instruments, Innsbruck, Austria) respirometer and fluorometer as previously described.^11^, ^19^, ^33^ Briefly, OCR and hydroperoxide production were measured in permeabilized fibre bundles in buffer Z media containing 10 μM Amplex UltraRed (Molecular Probes, Eugene, OR), 1 U/mL horseradish peroxidase, superoxide dismutase, and blebbistatin (25 μM) at 37°C. Rates of respiration and hydroperoxide production were determined using the following sequential additions of substrates and inhibitors: glutamate (10 mM), malate (2 mM), pyruvate (5 mM), Adenosine diphosphate (5 mM), succinate (10 mM), rotenone (1 μM), antimycin A (1 μM), and N,N,N′,N′‐Tetramethyl‐p‐phenylenediamine (TMPD) (0.5 mM) immediately followed by ascorbate (5 mM, ascorbate is added to ensure TMPD is reduced, so TMPD can continue to donate electrons). Respiration measurements were normalized to antimycin A to account for non‐mitochondrial oxygen consumption. Data for both OCR and rates of hydroperoxide generation were normalized by milligrams of muscle bundle wet weights weighed on Acculab AL‐104 scale.

### RNA isolation, cDNA synthesis, and quantitative real‐time PCR

Gastrocnemius muscles were collected and frozen in liquid nitrogen at time of harvest. Gastrocnemius muscle, 20–30 μg, was homogenized into a 1 mL TRIzol solution. RNA was isololated as previously described.^8^ Isolated RNA purity and concentration were confirmed using Bio‐Tek (Winooski, VT) Power Wave XS plate reader with Take3 microvolume plate and Gen5 software. After which, 1 μg of RNA was reverse transcribed into cDNA using previously described methods^8^ and iScriptTM cDNA Synthesis reverse transcriptase reagents. cDNA was diluted to 1:25 (25 ng/μL) and Ct values analysed using Sybr Green reagents and commercial QuantStudio 6 Flex real‐time RT‐PCR instrumentation (Applied BioSystems, Foster City, CA). The following primers were used for RT‐PCR assessment (*Table* S1): *18s*, *ND4*, *ND6*, *COX1*, *CytB*, *ATPase 8/6*, *ATP5F1*, *SDHA*, and *UQCRC1*. No differences were seen in *18s* among experimental conditions for experiments presented. Final quantification of gene expression was calculated using the ΔΔCT method. Relative quantification was calculated as 2−ΔΔCT.

#### Immunoblotting

Immunoblot was performed as previously described.^47^ Briefly, gastrocnemius muscle was homogenized in a buffer containing 0.23 M Tris HCl, pH 6.8, 4.5% *w*/*v* SDS, 45% glycerol, 0.04% *w*/*v* bromophenol blue, 80 mM dithiothreitol, 0.57 mM 2‐mercaptoethanol, protease inhibitor and denatured at 95°C. Concentrations were determined using the RC/DC assay (500‐0119, BioRad, Hercules, CA), and 30 μg total protein was resolved by sodium dodecyl sulfate‐polyacrylamide gel electrophoresis, transferred to a nitrocellulose membrane and blocked in 5% weight by volume milk in Tris‐buffered saline with 0.2% Tween 20. Membranes were probed overnight for antibodies specific to CuZn Superoxide Dismutase (SOD), MnSOD, Voltage‐dependent anion channel (VDAC), Cytochrome C Oxidase Subunit IV (CoxIV), and Thioredoxin‐dependent peroxide reductase (Prdx3). Primary and secondary antibodies were diluted in Tris‐buffered saline with 0.2% Tween 20 and used according to manufacturer's protocol. Membranes were imaged using Syngene G Box. All bands were normalized to the 45 kDa actin band of Ponceau S stain as a loading control.

### Sciatic nerve conduction velocity

Sciatic nerve conduction velocity was measured based on a previously described protocol.^48^ Briefly, mice were anaesthetised with constant flow of isoflurane. Sciatic nerve conduction velocity was measured using stimulating electrodes placed at the ankle, and recording electrodes were placed dorsally over all five digits. The latency and distance between electrodes were measured, and then, the stimulating electrodes were moved to the sciatic notch. The nerve was again stimulated, and the resulting latency was subtracted from the initial ankle–foot latency. This difference was divided between the distance between the notch and ankle to determine velocity. The distance was determined by stretching the foot so that a linear distance could be measured between stimulating and recording electrodes.

### Transmission electron microscopy for sciatic nerve

Sciatic nerve samples were immediately collected after sacrifice, fixed in 4% glutaraldehyde and post‐fixed 1% osmium. The Oklahoma Medical Research Foundation imaging core facility then processed the sciatic nerves for ultrastructure assessment. Images were taken on a Hitachi H‐7600 Transmission Electron Microscope at ×1200 magnification.

### Enzyme activity assay

Activities of CuZnSOD and MnSOD were determined using native gels with negative staining as a method previously described.^49^ Briefly, Extracts containing 40 μg protein were separated on a 10% polyacrylamide native page gel. The gel was then soaked in a solution containing nitroblue tetrazolium, riboflavin, and Tetramethylethylenediamine (TEMED). The riboflavin is activated to oxidize an electron donor (TEMED). The gel was then imaged using Syngene G Box. For publication purposes, the image was inverted so that the achromatic areas representing SOD activity appear as dark regions against a light background.

### Statistical analysis

A two‐way ANOVA with the independent factors of genotype and LLC implantation was used as the global analysis for each dependent variable. Only when there were differences was the Tukey–Kramer post hoc test performed. For all experiments, the comparison‐wise error rate, *α*, was set at 0.05 for all statistical tests. Asterisk (*) was used to denote significant differences denoted from the post hoc test. Interactions were denoted by #. All data were analysed, and graphs were compiled using GraphPad Prism (La Jolla, CA, USA) and data expressed as mean ± SEM.

## Results

### Characterization of Lewis lung carcinoma‐induced cancer cachexia in wild‐type and Sod1KO mice

**Figure 1:**
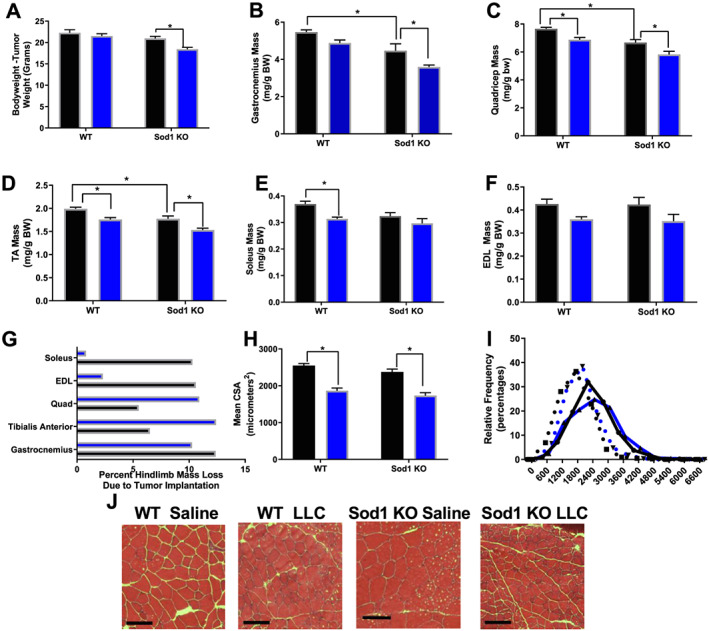
Characterization of LLC‐induced cancer cachexia in wild‐type (WT) and Sod1KO mice. (A) Body weights (BW—tumour weight) of WT saline, WT Lewis lung carcinoma (LLC), Sod1KO saline, and Sod1KO LLC tumour‐bearing mice. (B) Gastrocnemius mass in all groups normalized to BW. (C) Quadricep mass in all groups normalized to BW. (D) Tibialis anterior (TA) mass in all groups normalized to BW. (E) Soleus mass in all groups normalized to BW. (F) Extensor digitorum longus (EDL) mass in all groups normalized to BW. (G) Percent mass lost from cancer in hindlimb muscles of WT and Sod1KO mice. (H) Mean cross‐sectional area (CSA) analysis for all groups. (I) Frequency distribution of small fibres, average fibres, and large fibres. Groups for frequency distribution are displayed as shown here. (J) Representative images for haematoxylin and eosin stain across all groups. Scale bar is 100 μM. *N* of 6–8 was used for each group. Saline, black bar; LLC, blue bar. Asterisk denotes post hoc differences at an alpha set at *P* < 0.05.

In order to determine if elevated oxidative stress exacerbates cancer cachexia, we measured body weight, muscle weights, and cross‐sectional area (CSA) of hindlimb muscles. Injection of Sod1KO mice with LLC caused a 15% reduction in body weight compared with Sod1KO saline (*P* = 0.0107), suggesting that cancer‐induced loss of body weight was more severe in Sod1KO mice when compared with WT mice (*Figure* 1A). Gastrocnemius, quadriceps femoris, and TA muscle wet weights normalized to body mass were ~20% smaller in Sod1KO mice when compared with WT mice (*P* = 0.005–0.01, *Figure* 1B–D). Gastrocnemius mass was ~20% smaller in tumour‐bearing Sod1KO mice than gastrocnemius mass of saline‐injected Sod1KO mice (*P* = 0.017), which indicates that tumour‐mediated gastrocnemius muscle loss was more severe in Sod1KO mice than WT mice (*Figure* 1B). Quadricep femoris and TA muscle wet weights decreased by ~15% in tumour‐bearing WT and Sod1KO mice compared with saline‐injected WT and Sod1KO mice (*P* = 0.001–0.03, *Figure* 1C and 1D). Soleus mass was ~15% smaller in tumour‐bearing WT mice than saline‐injected WT mice (*P* = 0.019, *Figure* 1E). EDL mass was not different between groups (*Figure* 1F). The degree of tumour‐induced loss of muscle wet weight in hindlimb muscles is different in WT and Sod1KO mice (*Figure* 1G).

Despite a reduction in muscle mass in Sod1KO mice compared with WT mice, the mean CSA of quadricep femoris muscle fibres in the muscles was not different than in WT mice (*Figure* 1H). The mean CSA of quadricep femoris muscle fibres was ~25% smaller in tumour‐bearing mice when compared with saline‐injected WT and Sod1KO mice (*P* = 0.001, *Figure* 1H and 1J). The number of small fibres (200–1200 μm^2^ area) increased in tumour‐bearing WT and Sod1KO mice when compared with saline‐injected WT and Sod1KO mice and the number of large fibres (>2400 μm^2^) decreased in tumour‐bearing WT and Sod1KO mice when compared with saline‐injected WT and Sod1KO mice (*Figure* 1I and 1J). Both the mean quadriceps femoris CSA and the frequency distribution suggest that cancer‐induced loss of individual muscle fibre mass was not exacerbated in Sod1KO mice (*Figure* 1H and 1I). *Figure* 1J shows representative haematoxylin and eosin staining images from the quadriceps femoris used to assess CSA.

### Oxidative stress‐induced contractile dysfunction was not exacerbated in tumour‐bearing mice

**Figure 2:**
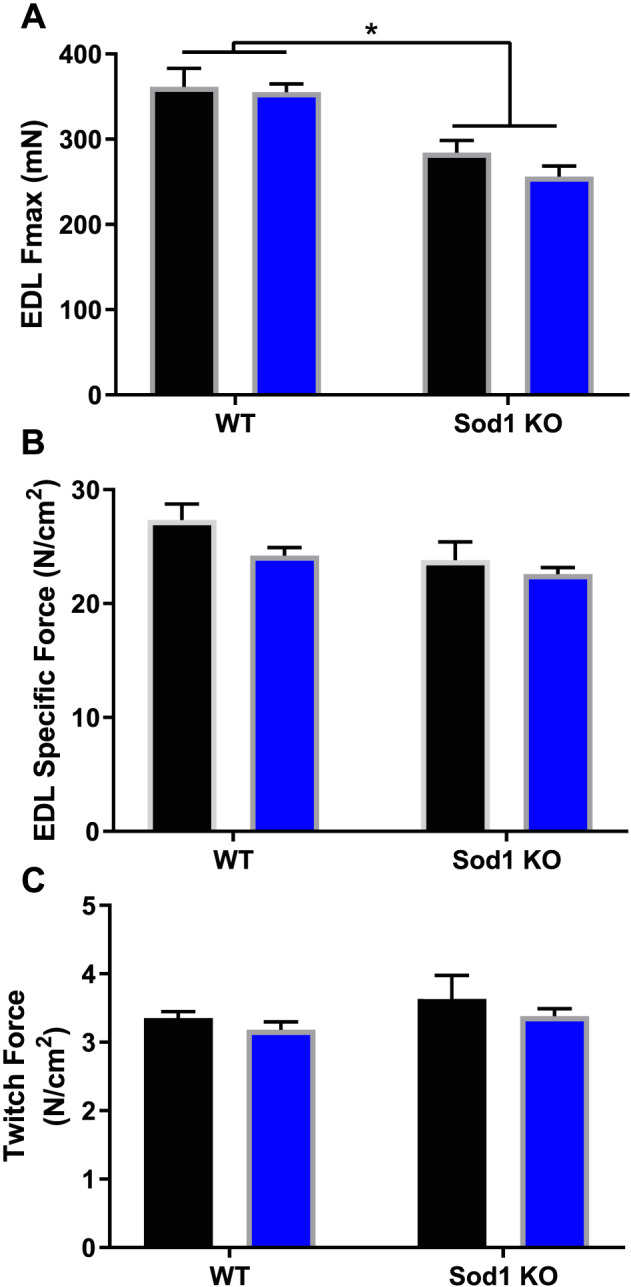
Oxidative stress‐induced contractile dysfunction is not exacerbated in tumour‐bearing mice. (A) Twitch contractile force for EDL muscle ex *vivo*. (B) Maximal contractile force for EDL muscle *ex vivo*. (C) Specific maximal contractile force for EDL muscle *ex vivo*. Saline, black bar; Lewis lung carcinoma, blue bar. Asterisk denotes post hoc differences at an alpha set at *P* < 0.05. EDL, extensor digitorum longus; WT, wild type.

In agreement with our previous reports, EDL maximal force was reduced by ~25% in Sod1KO mice when compared with WT counterparts (*P* = 0.012, *Figure* 2A). Despite a mean decrease of specific force in Sod1KO mice, there was no significant decrease between groups (*P* = 0.15, *Figure* 2B). We analysed the *ex vivo* twitch force of the EDL muscle and found no difference between groups (*Figure* 2C). Interestingly, the presence of tumours did not exacerbate contractile dysfunction in Sod1KO mice (*Figure* 2A–C).

### Tumour burden in Sod1KO mice exacerbates neuromuscular junction fragmentation

**Figure 3:**
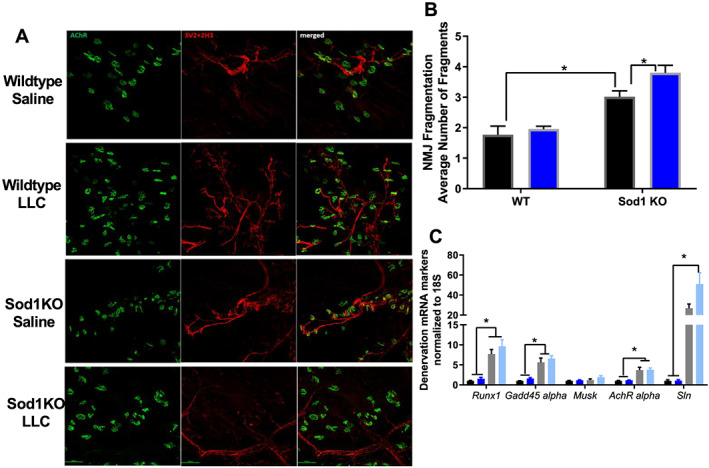
Cancer does not exacerbate neuromuscular junction disruption. (A) Representative images for NMJ staining in all groups. (B) NMJ fragmentation in all groups. Saline, black bar; LLC, blue bar. (C) Colours for graphs displayed in (C) are displayed here. Wild‐type saline, black bar; wild‐type LLC, dark blue bar; Sod1KO saline, grey bar; Sod1KO LLC, light blue bar. Denervation mRNA markers in all groups. For NMJ staining, an *N* of 2 animals per group was used. *N* of 6–8 per group was used in mRNA denervation markers.. Number sign denotes an interaction. Asterisk denotes post hoc differences at an alpha set at *P* < 0.05. LLC, Lewis lung carcinoma; NMJ, neuromuscular junction; WT, wild type.

Neuromuscular junction fragmentation increased by ~30% in saline‐injected Sod1KO mice when compared with WT saline mice (*P* = 0.03, *Figure* 3A and 3B). NMJ fragmentation was not different in tumour‐bearing WT mice compared with saline‐injected WT mice. NMJ fragmentation increased by an additional ~15% in tumour‐bearing Sod1KO mice when compared with saline‐injected Sod1KO mice (*P* = 0.01, *Figure* 3A and 3B). Sod1KO mice had a ~50–200% increase in mRNA content for the denervation markers *Runx1*, *Gadd45α*, *AchRα*, and *Sln* when compared with WT mice (*P* = 0.0001–0.0087, *Figure* 3C). Denervation markers did not increase in tumour‐bearing WT mice (*Figure* 3C), and tumour burden in Sod1KO mice did not further increase mRNA denervation markers when compared with saline‐injected Sod1KO mice (*Figure* 3C).

### Metabolic enzymes are altered in both Sod1KO mice and tumour‐bearing mice

**Figure 4:**
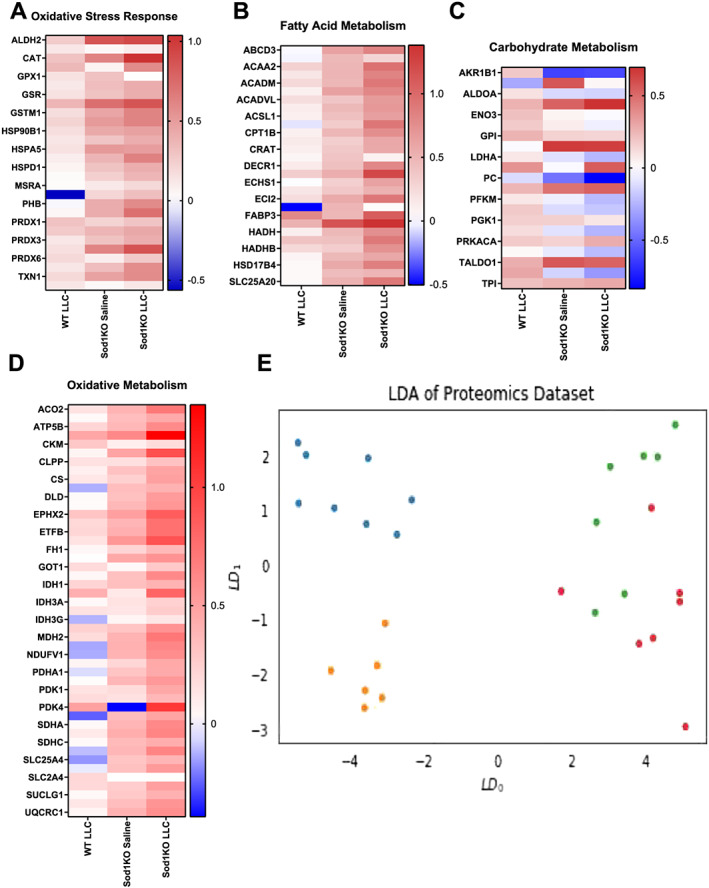
Metabolic enzymes are altered in Sod1KO mice and tumour‐bearing mice. (A) Heat map for oxidative stress enzymes relative to wild‐type saline mice. (B) Heat map for fatty acid metabolism enzymes relative to wild‐type saline mice. (C) Heat map for carbohydrate metabolism enzymes relative to wild‐type saline mice. (D) Heat map for oxidative metabolism enzymes relative to wild‐type saline mice. Targeted proteomics of glycolysis enzymes in all groups. (E) LDA analysis for the proteomics data set. Red, wild‐type saline; green, wild‐type LLC; orange, Sod1KO saline; blue, Sod1KO LLC. *N* of 6–8 per group was used. LDA, linear discriminant analysis; LLC, Lewis lung carcinoma; WT, wild type.

We were interested in how metabolic enzymes were altered in WT mice and Sod1KO mice with cancer. In general, the gastrocnemius muscle from Sod1KO mice showed an up‐regulation of proteins involved in oxidative stress response, fatty acid metabolism, and oxidative metabolism when compared with saline‐injected WT mice (*Figure* 4A–D). Carbohydrate metabolism proteins were both up‐regulated and down‐regulated in Sod1KO mice compared with saline‐injected WT mice (*Figure* 4C). Tumour burden had a more modest effect on proteins involved in oxidative stress response and fatty acid metabolism when compared with saline‐injected WT mice (*Figure* 4A and 4B); however, proteins involved in carbohydrate metabolism were largely up‐regulated (*Figure* 4C). Oxidative metabolism proteins were both up‐regulated and down‐regulated in tumour‐bearing WT mice when compared with saline‐injected WT mice (*Figure* 4D). Metabolic protein dysregulation in tumour‐bearing Sod1KO mice was exacerbated when compared with saline‐injected Sod1KO mice (*Figure* 4A–D). LDA analysis, a linear transformation technique that attempts to find a feature subspace that maximizes group separability, showed distinct separation between the metabolic proteins of WT and Sod1KO mice (*Figure* 4E). There was no distinct separation between saline‐injected WT mice and tumour‐bearing WT mice (*Figure* 4E). Metabolic profile of tumour‐bearing Sod1KO mice was different than saline‐injected Sod1KO mice (*Figure* 4E). Statistics for these proteins are shown in *Table* S1.

### Tumour‐bearing mice have protein imbalance favouring degradation, despite an oxidative stress‐associated increase in myofibrillar protein synthesis

**Figure 5:**
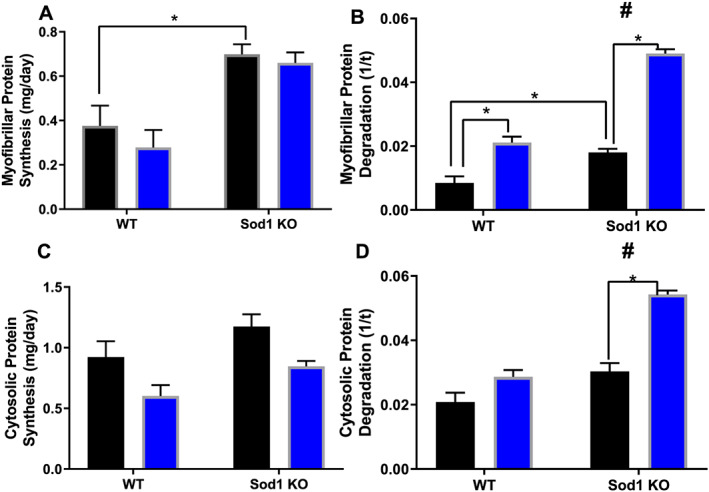
Tumour‐bearing mice have protein imbalance favouring degradation, despite an oxidative stress associated increase in myofibrillar protein synthesis. (A) Myofibrillar protein synthesis between all groups. (B) Myofibrillar protein degradation between all groups. (C) Cytosolic protein synthesis between all groups. (D) Cytosolic protein degradation between all groups. Saline, black bar; Lewis lung carcinoma, blue bar. Number sign denotes if there was an interaction. Asterisk denotes post hoc differences at an alpha set at *P* < 0.05. WT, wild type.

Muscle mass is lost when protein breakdown exceeds protein synthesis; therefore, we used D_2_O labelling to measure protein turnover in the TA muscle of WT and Sod1KO mice in response to induction of cachexia. Myofibrillar protein synthesis was two‐fold greater in saline Sod1KO mice when compared with saline WT mice (*P* = 0.027, *Figure* 5A). Myofibrillar degradation was two‐fold greater in saline Sod1KO mice than in saline WT mice (*P* = 0.0067, *Figure* 5B). Importantly, myofibrillar protein degradation was two‐fold greater in tumour‐bearing WT and Sod1KO mice when compared with WT saline and Sod1KO saline, respectively (*P* = 0.0001–0.0007, *Figure* 5B). Tumour‐bearing Sod1KO mice had ~50% greater increase in myofibrillar degradation rates when compared with tumour‐bearing WT mice (*P* = 0.0001, *Figure* 5B). Cytosolic proteins are typically involved in cell signalling and regulatory functions. There was no change in cytosolic protein synthesis between groups (*Figure* 5C). Cytosolic protein degradation did not increase in tumour‐bearing WT mice when compared with saline‐injected WT mice; however, cytosolic protein degradation was ~100% greater in tumour‐bearing Sod1KO mice when compared with saline Sod1KO mice (*P* = 0.0046, *Figure* 5D).

### Tumour‐bearing mice have impaired mitochondrial function, increased reactive oxygen species production, increased mitochondrial protein degradation, and a loss of oxidative fibres

**Figure 6:**
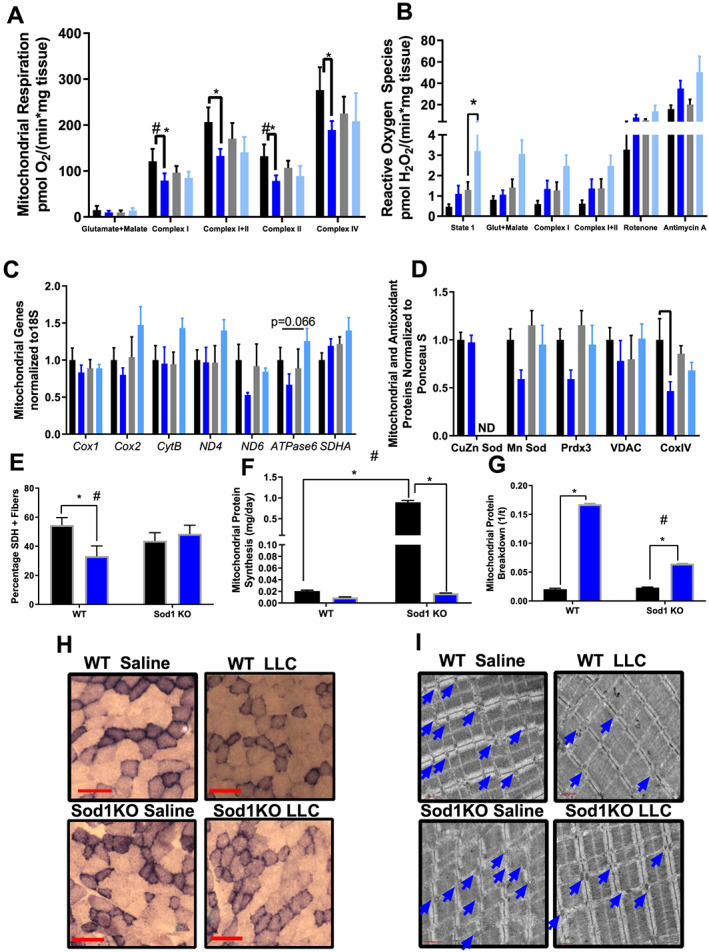
Tumour‐bearing mice have impaired mitochondrial function, increased reactive oxygen species production, and a loss of oxidative fibres. (A) Respiration measurements normalized to muscle wet weight. (B) Peroxide assessments normalized to muscle wet weight. There were main effects for both the genotype and LLC for an increase in hydroperoxide production for state 1, glutamate malate, complex I, complex I + II, and complex II stimulated ROS production. There was an ME for cancer‐induced increased in ROS production after antimycin A electron transport chain inhibition. Colours for graphs (C–E) are displayed here. (C) mRNA content of mitochondria genes. (D) Protein content of mitochondrial and antioxidant proteins. (E) Percent area stained positive for SDH. (F) Mitochondrial protein synthesis measurement using CoxIV as a mitochondrial content marker (Figure 6D). (G) Mitochondrial protein breakdown measurement using CoxIV as a mitochondrial content marker (Figure 6D). (H) SDH stain representative images. (I) TEM representative images. For TEM imaging, an *N* of 2 per group was used. *N* of 6–8 per group was used for all other measurements. (A–D) Wild‐type saline, black bar; wild‐type LLC, dark blue bar; Sod1KO saline, grey bar; Sod1KO LLC, light blue bar. (E–G) Saline, black bar; LLC, blue bar. Number sign denotes an interaction. Asterisk denotes post hoc differences at an alpha set at *P* < 0.05. LLC, Lewis lung carcinoma; WT, wild type.

Mitochondrial dysfunction is prevalent in multiple muscle wasting conditions including cancer cachexia^19^; therefore, we wanted to determine if elevated oxidative stress exacerbates cancer‐induced mitochondrial dysfunction and cancer‐induced mitochondrial turnover dysregulation. We assessed mitochondrial respiration and hydroperoxide production using permeabilized muscle fibres. Glutamate and malate‐stimulated leak respiration was not different between groups. Next, we measured maximally stimulated respiration from electron transport chain complex I, complex I + II, and complex II. These measurements assess mitochondrial peak respiratory capacity (state 3). There was a trend for a ~20% mean decrease (non‐significant, *P* = 0.1–0.25) in maximally stimulated respiration (complex I, complex I + II, and complex II) in Sod1KO when compared with WT counterparts (*Figure* 6A). Complex I, complex I + II, and complex II‐stimulated state 3 respiration was ~30% lower in tumour‐bearing WT mice when compared with saline‐injected WT mice (*P* = 0.0002–0.0023, *Figure* 6A). To assess the function of mitochondrial complex IV, we stimulated mitochondria with ascorbate and TMPD, which directly feeds elections into complex IV. Complex IV‐stimulated respiration was ~20% lower in tumour‐bearing WT mice than saline‐injected WT mice (*P* = 0.0074, *Figure* 6A). Taken together, these data suggest that cancer disrupts maximal mitochondrial respiratory, and mitochondrial dysfunction in tumour‐bearing Sod1KO mice was not further impaired (*Figure* 6A).

When mitochondria become damaged, electrons are more susceptible to leak into oxygen to form ROS. We wanted to determine if elevated oxidative stress triggers an increase in ROS production with cancer. State 1 hydroperoxide production was ~200% higher in tumour‐bearing Sod1KO mice when compared with saline‐injected Sod1KO mice (*P* = 0.033, *Figure* 6B). Increased state 1 hydroperoxides is typically associated with muscle that has lost innervation,^50^, ^51^ which corroborates our findings in *Figure* 3. ROS generation during maximally stimulated respiration is indicative of electrons prematurely leaking into oxygen. There were no differences in ROS production between groups with complex I, complex I + II, and complex II‐stimulated respiration (*Figure* 6B). Rotenone leads to reverse electron flow and leak at complex I while antimycin A causes electron backup and leak for the entire electron transport chain. There were no differences in ROS production between groups following rotenone and antimycin A treatment (*Figure* 6B). These data suggest that cancer exacerbates basal hydroperoxide production in Sod1KO mice; however, in stimulated conditions, hydroperoxides produced in tumour‐bearing Sod1KO mice are not higher than saline‐injected Sod1KO mice.

Considering the loss of mitochondrial respiration in tumour‐bearing mice, we were interested in the effect of the tumour on mitochondrial turnover and content in skeletal muscle because impaired mitochondrial turnover can lead to mitochondrial dysfunction. For these calculations, we used the mitochondrial content marker CoxIV (*Figure* 6D). mRNA content for mitochondrial genes were not different between groups (*Figure* 6c); however, mitochondrial proteins MnSOD, Prdx3, and CoxIV were decreased by ~20–50% in WT and Sod1KO tumour‐bearing mice when compared with WT saline and Sod1KO saline mice, respectively (*P* = 0.02–0.049, *Figure* 6D). Immunoblot images are shown in *Figure* S1. The percentage of the image area that stained for SDH was not different between genotypes (*Figure* 6E and 6H). However, the area stained in tumour‐bearing WT mice was ~35% lower than saline‐injected WT mice (*P* = 0.042, *Figure* 6E and 6H). Loss of stained SDH area was not different in LLC‐injected Sod1KO mice when compared with saline‐injected Sod1KO mice (*Figure* 6E and 6H). Mitochondrial protein synthesis increased by ~300% in saline‐injected Sod1KO mice compared with all other groups (*P* = 0.0001, *Figure* 6F). Mitochondrial protein degradation increased by four‐fold in tumour‐bearing WT mice compared with saline‐injected WT mice (*P* = 0.0001, *Figure* 6G). Mitochondrial degradation in tumour‐bearing Sod1KO mice increased by two‐fold compared with saline‐injected Sod1KO mice, which was a notably smaller increase than tumour‐bearing WT mice (*P* = 0.0001, *Figure* 6G). Transmission electron microscopy (TEM) showed drastically increased size and quantity of mitochondria in Sod1KO mice compared with WT mice, while mitochondria of tumour‐bearing mice were smaller and less dense than saline‐injected mice (*Figure* 6I).

### Motor neuron dysfunction was present in Sod1KO mice and tumour‐bearing mice

**Figure 7:**
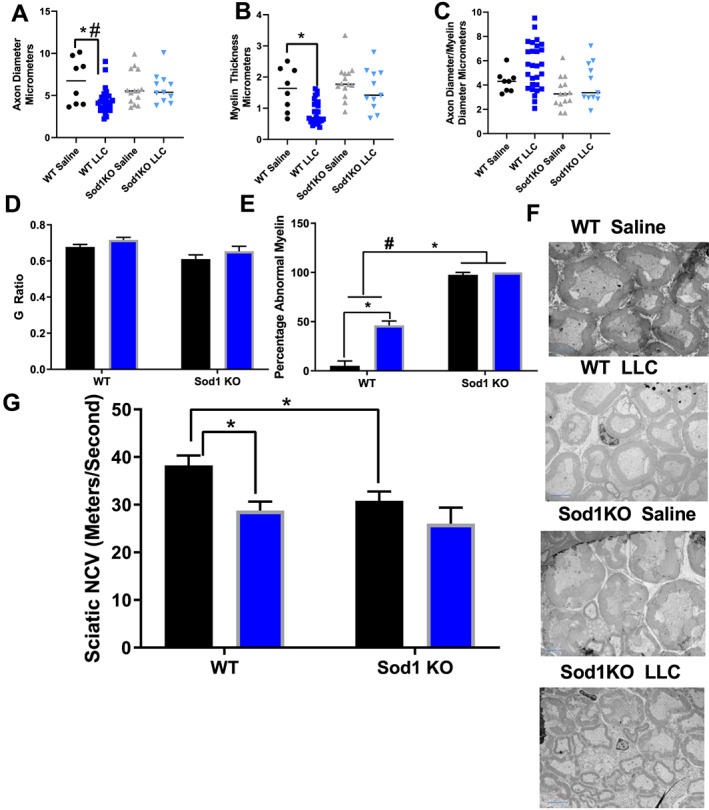
Motor neuron dysfunction is present in Sod1KO mice and tumour‐bearing mice. (A) Axon diameter measurements from sciatic nerve TEM images in all groups. (B) Myelin diameter measurements from sciatic nerve TEM images in all groups. (C) Axon diameter/myelin diameter from sciatic nerve TEM images in all groups. (D) *G* ratio from sciatic nerve TEM images in all groups. (E) Percent abnormal myelin from sciatic nerve TEM images in all groups. (F) Representative sciatic nerve TEM images. (G) Sciatic nerve conduction velocity assessment in all groups. For TEM image analysis, an *N* of 1 animal per group was used. *N* of 6–8 per group was used for nerve conduction velocity. (A–C) Wild‐type saline, black circles; wild‐type LLC, dark blue squares; Sod1KO saline, grey triangles; Sod1KO LLC, light blue inverted triangles. (D–G) Saline, black bar; LLC, blue bar. Number sign denotes an interaction. Asterisk denotes post hoc differences at an alpha set at *P* < 0.05. LLC, Lewis lung carcinoma; WT, wild type.

Sod1KO mice show a number of motor neuron phenotypes including demyelination, reduced nerve conduction velocity, and NMJ disruption.^29^, ^48^, ^52^, ^53^ Here, we asked whether the presence of tumours can exacerbate these phenotypes. The axon diameter measured in the sciatic nerve from Sod1KO mice was not different than in WT counterparts; however, axon diameter decreased by ~50% in tumour‐bearing WT mice compared with saline‐injected WT mice (*P* = 0.014, *Figure* 7A and 7F). Injection of LLC cells had no effect on axon diameter in Sod1KO mice (*Figure* 7A and 7F). There was no change in myelin diameter in Sod1KO mice compared with WT mice; however, myelin diameter decreased by ~40% in tumour‐bearing WT mice compared with saline‐injected WT mice (*P* = 0.0048, *Figure* 7B and 7F). Myelin diameter was not different in tumour‐bearing Sod1KO mice when compared with saline‐injected Sod1KO mice (*Figure* 7B and 7F). The axon diameter/myelin diameter and *G* ratio were not different between groups (Figure 7C, 7D, and 7F). The percent abnormal myelin increased by 100% in Sod1KO mice when compared with WT mice (*P* = 0.0001 *Figure* 7E and 7F). WT tumour‐bearing mice had 50% altered myelin profile, while saline‐injected WT mice did not have an altered myelin profile (*P* = 0.0001, *Figure* 7E and 7F). Sciatic nerve conduction velocity was decreased by ~20% in Sod1KO mice when compared with WT mice (*P* = 0.0223, *Figure* 7G). Also, sciatic nerve conduction velocity is decreased by ~30% in tumour‐bearing WT mice compared with saline‐injected WT mic`e (*P* = 0.0038, *Figure* 7G). Sciatic nerve conduction velocity was not further decreased in tumour‐bearing Sod1KO mice (*Figure* 7G).

### Lewis lung carcinoma injection induced death of Sod1KO mice within three weeks

**Figure 8:**
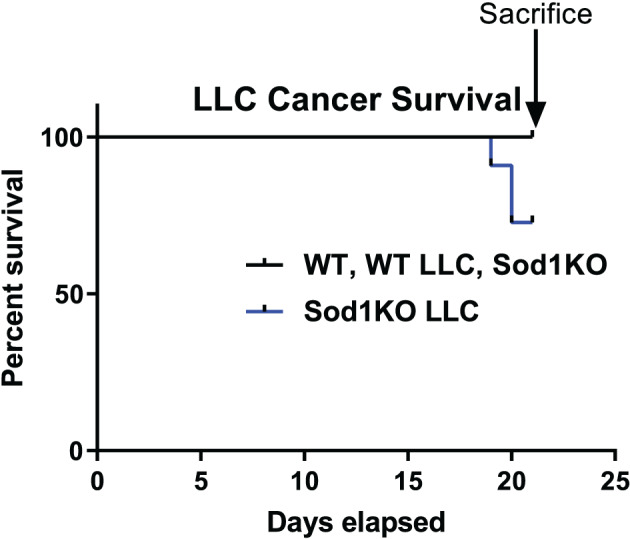
LLC‐injection induced death of Sod1KO mice within 3 weeks. Survival curve of tumour‐bearing WT (*n* = 8) and Sod1KO (*n* = 11) mice. Black line, wildtype saline, wildtype LLC, and Sod1KO saline. Blue line, Sod1KO LLC. LLC, Lewis lung carcinoma; WT, wild type.

We injected 4‐month to 5‐month‐old female WT and Sod1KO mice with phosphate‐buffered saline or one million LLC cells. Unexpectedly, 3 out of 11 of the tumour‐bearing Sod1KO mice did not survive the 3‐week duration of tumour implantation, while no WT mice that were injected with LLC cells died during the experimental period (*Figure* 8). Therefore, we ended the experiment after 3 weeks for data collection. Saline‐injected WT and Sod1KO mice survived the entire duration of the study as well (*Figure* 8).

## Discussion

It is currently unknown if increased oxidative stress exacerbates cancer cachexia. The primary findings of our study are that knocking out the *Sod1* gene, which codes for a critical antioxidant enzyme and superoxide anion scavenger, increases cancer mortality rates but does not exacerbate muscle loss, mitochondrial dysfunction, ROS production, or loss of oxidative phenotype in cachectic muscle in tumour‐bearing mice. Moreover, our study shows that protein turnover changes in a way that leads to a gain in mitochondrial mass in Sod1KO mice when compared with WT mice and that tumour‐bearing mice have increased protein degradation leading to a loss of mitochondrial content. We further found that induction of cancer exacerbates metabolic enzyme dysregulation and NMJ fragmentation in Sod1KO mice.

The pre‐clinical model we have elected to use in our study is implanting LLC cells (1 × 10^6^ cells) into the hind flank of mice. This model has been used by multiple laboratories to induce cancer cachexia.^54^, ^55^, ^56^ Several laboratories have noted that implanting 1 × 10^6^ LLC cells into the hind flank of mice induces a moderate to severe cancer cachexia phenotype (~20% body mass lost and ~30–45% gastrocnemius muscle mass lost) after approximately 30 days of tumour growth.^54^, ^55^, ^56^ In our model, we measured muscle mass loss 3 weeks after tumour implantation because of the abrupt deaths experienced in the Sod1KO mice at that time point. Both WT and Sod1KO mice display a similar degree of muscle wet weight and CSA (~10% smaller muscle wet weights and a ~25% decrease in mean CSA). We would consider this a mild cancer cachexia phenotype based on more severe phenotypes commonly being observed in other studies and models^57^, ^58^, ^59^; however, we could not extend the study longer based on the survival of the Sod1KO mice. Further, female mice are less susceptible to cancer cachexia in pre‐clinical models and in cachectic patients,^60^, ^61^, ^62^ which could be another reason why our phenotype is not as severe as other studies. Despite a 20% decrease in muscle mass in Sod1KO mice, fibre CSA was not different when compared with saline‐injected WT mice. This would suggest that the smaller muscle size observed in Sod1KO mice at this age is primarily due to loss of fibre number, which has previously been reported in the Sod1KO mouse model.^63^, ^64^ To our surprise, tumour implantation did not decrease EDL contractility in either WT or Sod1KO mice. It is possible that the discrepancies between our data and other cancer cachexia contractility reports are due to the severity of phenotype observed in these studies and the fibre type of the muscle used.^57^, ^65^ Based on these results, elevated oxidative stress may not exacerbate cancer‐related loss of muscle mass and function; however, if cancer cachexia was allowed to further progress, it is possible that loss of *Sod1* could exacerbate the phenotype at a later time point.

Skeletal muscle atrophy occurs by an imbalance of myofibrillar protein turnover favouring protein degradation over protein synthesis. According to our data, tumour‐bearing mice have increased degradation in myofibrillar fractions and decreased protein synthesis in cytosolic fractions. To our knowledge, this is the first time long‐term protein degradation has been assessed (rather than markers) in a tumour model. It should also be noted that rates of myofibrillar protein degradation were higher in tumour‐bearing Sod1KO mice when compared with tumour‐bearing WT mice. However, this did not exacerbate the cancer cachexia phenotype in Sod1KO mice possibly due to higher rates of myofibrillar protein synthesis. The Sod1KO mouse is used as an accelerated aging and frailty model,^9^ and similar to the Sod1KO mouse model, myofibrillar protein synthesis is elevated in aged muscle.^66^ Prior studies show that atrogenes and autophagy machinery is up‐regulated in tumour‐bearing mice, which corresponds with our direct measurement of protein breakdown.^67^, ^68^, ^69^ Further, proteolytic markers are elevated in the Sod1KO mouse model,^29^ so this may contribute to our direct measurement showing increased protein degradation in tumour‐bearing Sod1KO mice even when compared with tumour‐bearing WT mice. It is possible that pathways that contribute to protein breakdown are even further elevated in tumour‐bearing Sod1KO mice. Our protein turnover measurements in myofibrillar and cytosolic fractions highlight the need to assess both aspects of dynamic protein turnover (not markers) during non‐steady state conditions such as cancer cachexia, which allows for a more robust interpretation than just measurements of protein turnover with markers.

Mitochondria are critical for the maintenance of muscle mass and function^70^, ^71^, ^72^; therefore, we examined skeletal muscle mitochondrial properties and hydroperoxide production in tumour‐bearing WT and Sod1KO mice. Maximally stimulated respiration (state 3 respiration) was reduced in cachectic muscle in WT mice; however, cancer did not alter mitochondrial respiration in Sod1KO mice. Also, we observed increased ROS production ranging from 10% to 200% based on the substrate/inhibitor used. It is well established that mitochondrial damage/dysfunction and chronically elevated ROS generation lead to muscle wasting.^73^, ^74^, ^75^, ^76^, ^77^, ^78^, ^79^ Based on these results and previous findings, it is likely that mitochondrial damage is involved in cancer‐induced muscle loss. More research is required to show if mitochondrial dysfunction directly contributes to cancer cachexia.

Both total mitochondrial content and mitochondrial turnover are key drivers of respiratory capacity in skeletal muscle; therefore, we measured mitochondrial turnover in tumour‐bearing WT and Sod1KO mice. Loss of oxidative phenotype (SDH stain) and mitochondrial content (Prdx3, MnSOD, and CoxIV) occurs in WT tumour‐bearing mice, but not tumour‐bearing Sod1KO mice, which may contribute to the loss of respiratory capacity in tumour‐bearing mice. Mitochondrial protein synthesis is dramatically up‐regulated in Sod1KO mice and cancer completely blunts this increase in mitochondrial protein synthesis. Also, mitochondrial protein degradation is dramatically elevated in tumour‐bearing mice. These data are corroborated by the increase in mitochondria size in muscle from Sod1KO mice and reduced mitochondria volume in tumour‐bearing mice shown via electron microscopy images. These findings are consistent with previous studies showing that there is a loss of the oxidative phenotype in skeletal muscle of tumour‐bearing mice.^18^, ^56^, ^80^

Because mitochondrial content is differentially affected by the Sod1KO mouse and tumour‐bearing mice, we used targeted proteomics to illustrate changes in metabolic and antioxidant proteins. Sod1KO mice have a global change in the protein content of metabolic enzymes and antioxidant enzymes, which is exacerbated in tumour‐bearing Sod1KO mice. Surprisingly, proteins involved in oxidative stress response are relatively unchanged in WT tumour‐bearing mice, which suggests that ROS was not chronically elevated enough for an oxidative stress response. However, oxidative stress response and dysregulation of metabolic enzymes are exacerbated in tumour‐bearing Sod1KO mice, showing that these mice may be more susceptible to tumour‐induced changes in metabolism.

Prior research from our laboratory clearly shows that muscle loss and contractile dysfunction can be driven by neuromuscular impairments; therefore, we assessed motor neuron function and NMJ integrity. There was a loss of myelin and axon diameter in tumour‐bearing mice and abnormal myelin profiles in Sod1KO mice. We also observed impaired sciatic nerve conduction velocity in both Sod1KO mice and tumour‐bearing mice, which indicates that nerve function is impaired. Motor neuron impairments have been shown to lead to muscle pathology.^81^, ^82^ This is especially prevalent in disorders such as Amyotrophic lateral sclerosis. At this point, it is not known if motor neuron dysfunction contributes to cancer‐induced muscle loss. Sod1KO mice have NMJ impairments that mirror what happens in aged mice.^83^ NMJ fragmentation was further elevated in tumour‐bearing Sod1KO mice when compared with saline‐injected Sod1KO mice, which indicates that cancer could exacerbate NMJ impairments. These data would suggest that cancer in aged individuals may accelerate NMJ fragmentation, which would lead to sarcopenia.

In summary, this is the first study to investigate if increased oxidative stress and frailty exacerbates cancer cachexia in tumour‐bearing mice. Cancer in Sod1KO mice, a mouse model for oxidative stress and sarcopenia, did not exacerbate muscle wasting in tumour‐bearing mice. Also, we have revealed that the deletion of *Sod1* does not exacerbate cancer‐mediated contractile dysfunction, cancer‐mediated mitochondrial dysfunction, and cancer‐mediated ROS production. Cancer differentially alters myofibrillar and mitochondrial degradation in Sod1KO mice when compared with WT mice. Also, we have found that cancer exacerbates metabolic enzyme dysregulation and NMJ fragmentation in Sod1KO mice. To our knowledge, this is the first study to show that cancer disrupts motor neuron function; however, it is not known if this contributes to muscle loss. Future studies should examine cancer cachexia in an aged population considering aging is the greatest risk factor for cancer.

## Conflict of interest

All authors declare no conflicts of interest.

## Ethical standards statement

Animal experiments were approved by the Institutional Animal Care and Use Committees and performed at the Oklahoma Medical Research Foundation.

## Supporting information


**Figure S1.** **Sample blots and sample activity gel.** A. Sample blots for the quantitation displayed in figure 5G. B. Sample image of the activity gel ran to confirm the genotype of mice. Representative MnSod and CuZnSod enzyme activity gel in muscle, liver and brain. N of 6–8 per group was used.

**Table S1:** Forward and Reverse Sequences for Sybr primers.

**Table S2:** Statistical analysis for targeted proteomics dataset using LDA.
