# Supplementary figures and images for: Cancer cachexia in a mouse model of oxidative stress
Source: J Cachexia Sarcopenia Muscle. 2020 Sep 12;11(6):1688–704. doi: 10.1002/jcsm.12615 (PMC7749559; doi:10.1002/jcsm.12615)

# Figure S1

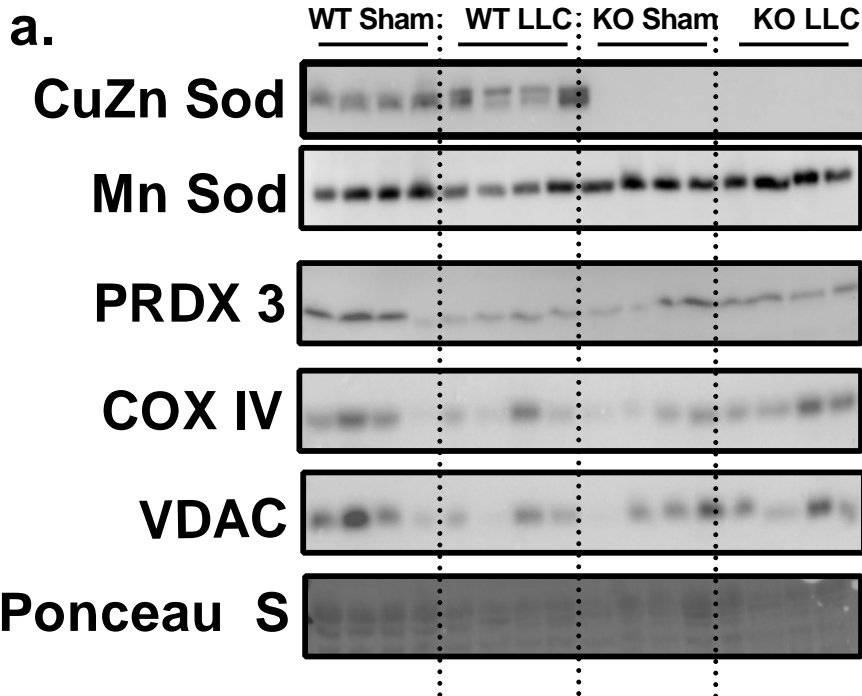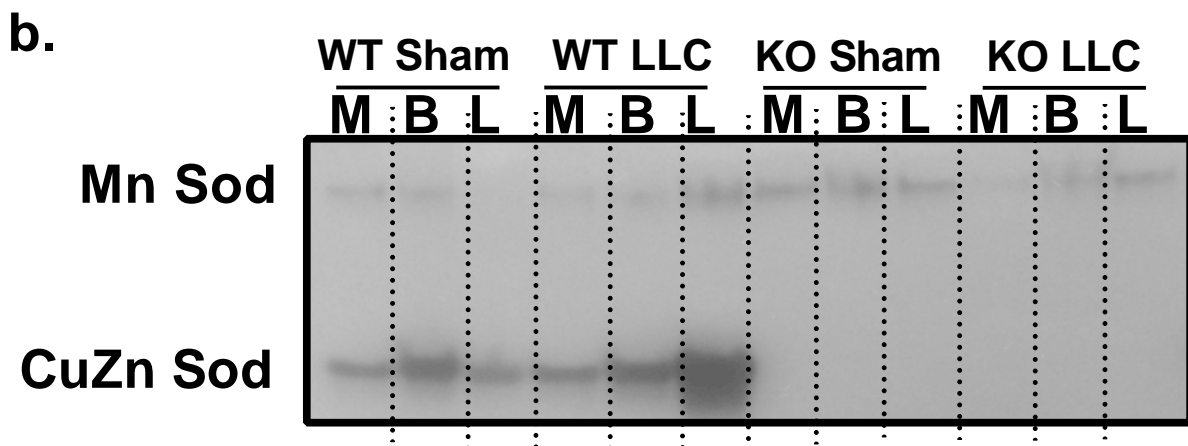

Supplement: Supplementary file 1 — Figure S1. Sample blots and sample activity gel. A. Sample blots for the quantitation displayed in figure 5G. B. Sample image of the activity gel ran to confirm the genotype of mice. Representative MnSod and CuZnSod enzyme activity gel in muscle, liver and brain. N of 6–8 per group was used. [file JCSM-11-1688-s001.pdf]
