# Supplementary material for: Cancer cachexia in a mouse model of oxidative stress
Source: J Cachexia Sarcopenia Muscle. 2020 Sep 12;11(6):1688–704. doi: 10.1002/jcsm.12615 (PMC7749559; doi:10.1002/jcsm.12615)
Supplement: Supplementary file 2 — Table S1: Forward and Reverse Sequences for Sybr primers. [file JCSM-11-1688-s002.docx]

**Table S1**

| ND4 | Forward | ACCTACTGGGAGAACTCTCTGT |
| --- | --- | --- |
|  | Reverse | GGTGAGTGAGCCCCATTGTGTT |
| ND6 | Forward | TGGGGTTAGCGATGGAGGTAGG |
|  | Reverse | AATAGGATCCTCCCGAATCAAC |
| COX1 | Forward | TGGAGGCTTTGGAAACTGAC |
|  | Reverse | TTCATCCTGTTCCTGCTCCT |
| COX2 | Forward | ATGGCCTACCCATTCCAACT |
|  | Reverse | CGGGGTTGTTGATTTCGTC |
| CytB | Forward | CTGATCCTCCAAATCACCACAG |
|  | Reverse | GCGCCATTGGCGTGAAGGTA |
| ATPase6 | Forward | ACACACCAAAAGGACGAACA |
|  | Reverse | GAAGGAAGTGGGCAAGTGAG |
| SDHA | Forward | CAGAAGTCGATGCAGAACCA |
|  | Reverse | CGACCCGCACTTTGTAATCT |
| Runx1 | Forward | GATGGCACTCTGGTCACCG |
|  | Reverse | GCCGCTCGGAAAAGGACAA |
| GADD45-α | Forward | AGACCGAAAGGATGGACACG |
|  | Reverse | GTACACGCCGACCGTAATG |
| MusK | Forward | TCCTGCGTGCTCCTGAATC |
|  | Reverse | TGCAGCGTAGGGTTACAAAGG |
| AchR-α | Forward | ACCTGGACCTATGACGGCTCT |
|  | Reverse | AGTTACTCAGGTCGGGCTGGT |
| SLN | Forward | GAGGTGGAGAGACTGAGGTCCTTGG |
|  | Reverse | GAAGCTCGGGGCACACAGCAG |
