# Supplementary material for: Cancer cachexia in a mouse model of oxidative stress
Source: J Cachexia Sarcopenia Muscle. 2020 Sep 12;11(6):1688–704. doi: 10.1002/jcsm.12615 (PMC7749559; doi:10.1002/jcsm.12615)
Supplement: Supplementary file 3 — Table S2: Statistical analysis for targeted proteomics dataset using LDA. [file JCSM-11-1688-s003.docx]

**Table S2**

|  | |  | t-statistic | p-value | p-adj |
| --- | --- | --- | --- | --- | --- |
| panel | | Protein |  |  |  |
| Stress Response | | SOD1 | 10.319047 | 1.10E-10 | 1.30E-08 |
| Carbohydrate Metabolism | AKR1B1 | | 7.493429 | 5.90E-08 | 3.48E-06 |
| Fatty Acid Metabolism | | FABP4 | -5.549793 | 7.92E-06 | 2.37E-04 |
|  | | ACADS | -5.544071 | 8.04E-06 | 2.37E-04 |
| Stress Response | | TXNRD1 | 5.096439 | 2.61E-05 | 5.54E-04 |
| Carbohydrate Metabolism | | PC | 5.024788 | 3.15E-05 | 5.54E-04 |
| Fatty Acid Metabolism | | ECI2 | -5.008637 | 3.28E-05 | 5.54E-04 |
|  | | SLC25A20 | -4.721392 | 7.01E-05 | 1.03E-03 |
|  | | ECH1 | -4.433847 | 1.50E-04 | 1.96E-03 |
| Carbohydrate Metabolism | | HK1 | -4.330255 | 1.97E-04 | 2.32E-03 |
| Oxidative Metabolism | | ETFDH | -4.239693 | 2.50E-04 | 2.68E-03 |
|  | | IDH3B | 4.078441 | 3.81E-04 | 3.75E-03 |
| Stress Response | | ALDH2 | -3.88584 | 6.29E-04 | 5.71E-03 |
| Oxidative Metabolism | | SLC2A4 | 3.743699 | 9.09E-04 | 7.66E-03 |
|  | | CD36 | -3.70957 | 9.92E-04 | 7.81E-03 |
|  | | SLC25A4/5/31 | -3.658304 | 1.13E-03 | 8.35E-03 |
| Stress Response | | PRDX5 | -3.608717 | 1.29E-03 | 8.92E-03 |
| Carbohydrate Metabolism | | PFKM | 3.487423 | 1.75E-03 | 1.15E-02 |
| Oxidative Metabolism | | IDH3A | 3.437097 | 1.99E-03 | 1.24E-02 |
|  | | SUCLA2 | 3.272164 | 3.01E-03 | 1.65E-02 |
| Fatty Acid Metabolism | | HSD17B4 | -3.271585 | 3.02E-03 | 1.65E-02 |
|  | | PECR | -3.263858 | 3.07E-03 | 1.65E-02 |
| Oxidative Metabolism | | FH1 | 3.218495 | 3.44E-03 | 1.77E-02 |
| Stress Response | | MSRA | 3.100947 | 4.60E-03 | 2.26E-02 |
| Carbohydrate Metabolism | | PGAM2 | 3.042834 | 5.30E-03 | 2.50E-02 |
|  | | PRKACA | 2.935759 | 6.87E-03 | 3.10E-02 |
| Oxidative Metabolism | | TUFM | 2.916822 | 7.20E-03 | 3.10E-02 |
| Fatty Acid Metabolism | | ACADL | -2.908212 | 7.35E-03 | 3.10E-02 |
| Carbohydrate Metabolism | | PGK1 | 2.765934 | 1.03E-02 | 4.11E-02 |
| Fatty Acid Metabolism | | ACADM | -2.759583 | 1.05E-02 | 4.11E-02 |
|  | | HADH | -2.707544 | 1.18E-02 | 4.43E-02 |
| Stress Response | | CAT | -2.700795 | 1.20E-02 | 4.43E-02 |
|  | | HSPD1 | 2.655682 | 1.33E-02 | 4.77E-02 |
| Oxidative Metabolism | | EPHX2 | -2.63424 | 1.40E-02 | 4.86E-02 |
